# Red Cross Conference in Cardiff

**Published:** 1920-10-30

**Authors:** 


### Red Cross Conference in Cardiff.

### PLANS OF THE JOINT ORGANISATION IN WALES.

The campaign of addresses which Sir Arthur Stanley, Chairman of the Joint Council, and Sir Napier Burnett, director of hospital services, gave throughout the country to secure co-operation between headquarters and local organisations in the peace programme of the British Red Cross Society and the Order of St. John, with special reference to "Our Day," proceeded right merrily. At Cardiff the two speakers met a distinguished gathering. The Lord Mayor, Mr. G. F. Forsdike, O.B.E., said that he trusted that the Welsh Joint Council would be given full self-controlling power.

Sir Arthur Stanley declared that Wales already had complete autonomy. It should be tied to headquarters by a slender thread, provided that thread was a strong one, and if it were a thread of gold so much the better. The war had welded the two bodies together, and that cordial relation had continued. No one had done more for it than the Earl of Plymouth, who placed himself at the head of the committee. There was still an immense amount of work to be done, which could be accomplished only if the societies worked together.

Sir Napier Burnett said that not even the Ministry of Health knew the number of crippled children in the country; yet most of those under five could be relieved of their disability. To find these cripples would be an admirable task for voluntary-aid workers. He then emphasised the value of district nursing, especially in country districts, where the nurses often brought the chief ray of sunshine known to the workers' homes. The joint societies might also open places where those run down in health could recuperate, and thus prevent much disease. He appealed to the joint committees to take up this work also.

The Earl of Plymouth alluded to Sir Arthur Stanley's services, and the value of his tact in overcoming difficulties. Though Wales liked to manage her own affairs, the face-to-face talk with representatives from headquarters was invaluable. He concurred in Sir Arthur's remarks on the possibility of establishing a joint council in Wales. In his own view, a Red Cross Branch Council in Wales, to confer with the Priory of the Order of St. John, would be a stronger body, selected chiefly from a group of counties more thickly populated than others. Organisation on a county basis could best be interpreted in this sense. They would loyally support the Joint Council.

